# Trial of an All-Ceramic SnO_2_ Gas Sensor Equipped with CaCu_3_Ru_4_O_12_ Heater and Electrode

**DOI:** 10.3390/ma11060981

**Published:** 2018-06-11

**Authors:** Akihiro Tsuruta, Toshio Itoh, Masashi Mikami, Yoshiaki Kinemuchi, Ichiro Terasaki, Norimitsu Murayama, Woosuck Shin

**Affiliations:** 1 National Institute of Advanced Industrial Science and Technology (AIST), Shimo-Shidami, Moriyama-ku, Nagoya 463-8560, Japan; itoh-toshio@aist.go.jp (T.I.); m-mikami@aist.go.jp (M.M.); y.kinemuchi@aist.go.jp (Y.K.); terra@cc.nagoya-u.ac.jp (I.T.); w.shin@aist.go.jp (W.S.); 2 Department of Physics, Nagoya University, Furo-cho, Chuikusa-ku, Nagoya 464-8602, Japan; 3 National Institute of Advanced Industrial Science and Technology (AIST), 1-1-1 Higashi, Tsukuba 305-8565, Japan; n-murayama@aist.go.jp

**Keywords:** conducting oxide, ceramics heater, gas sensor, perovskite

## Abstract

We have constructed a gas sensor of SnO_2_ equipped with ceramic electrodes and a heater made of CaCu_3_Ru_4_O_12_, which demonstrated good device performance at high temperature. The CaCu_3_Ru_4_O_12_-based electrodes and heater were formed on Al_2_O_3_ substrates using a screen-printing process, which is cost-effective and suitable for mass-production. This all-ceramic device reached 600 °C at the lowest, and remained intact after one week of operation at 500 °C and rapid thermal cycling of 500 °C temperature changes within 10 s. We propose CaCu_3_Ru_4_O_12_ as a robust and reliable conducting material that can be a substitute for Pt in various devices.

## 1. Introduction

Gas sensors used in fields including healthcare, fuel monitoring, and industrial gas production are under active study regarding sensing mechanisms, materials, and designs to realize high reliability, high-speed response, and miniaturization [1,2,3,4,5]. Although various sensing mechanisms are used in gas sensors, such as semiconductivity [6], thermoelectricity [7], and mixed-potential [8], high-temperature operation at several hundred degrees Celsius is necessary to improve the high-speed response and gas selectivity with any mechanism. For sensing in harsh gas atmospheres, such as high-concentration oxygen or fuel gases at high temperatures, Pt has been often used as a heater and electrode in such sensors owing to its chemical and thermal stability. Thus, the development of alternative materials for Pt is an important industrial issue that can drastically reduce the device cost.

**Figure 1:**
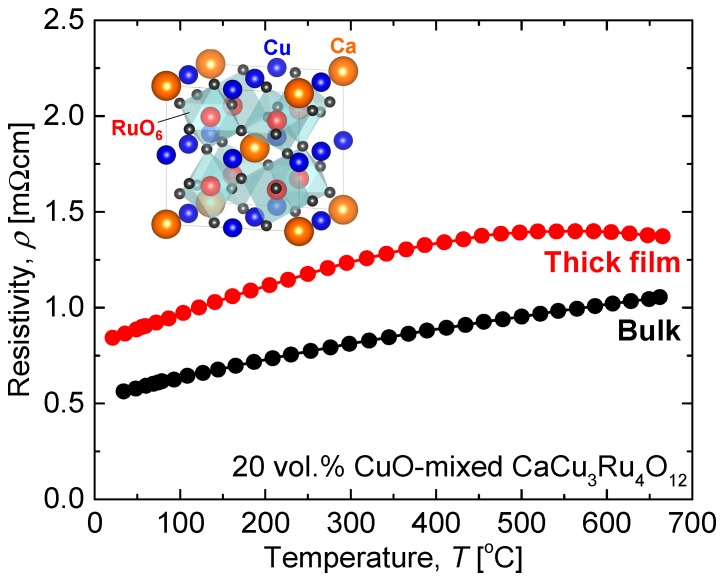
Temperature dependence of resistivity of 20 vol % CuO-mixed CaCu_3_Ru_4_O_12_ bulk and thick film. The inset is the crystal structure of CaCu_3_Ru_4_O_12_.

Oxides are promising class of materials that show environmental resistance and can be substitutes for Pt. However, few oxides exhibit high electrical conductivity with metallic temperature dependence, and state-of-the-art conducting oxides such as RuO_2_ [9], IrO_2_ [10], and ReO_2_ [11] are as expensive as Pt. We have focused on the ordered perovskite oxide CaCu_3_Ru_4_O_12_ [12,13] (the crystal structure is shown in the inset of Figure 1) and have studied its physical properties and practical processing [14,15,16]. The resistivity, temperature coefficient of resistance (TCR), and material cost are compared between Pt and CaCu_3_Ru_4_O_12_ in Table 1. The resistivity of CaCu_3_Ru_4_O_12_ is lower than 1 mΩ·cm even at 500 °C, and the cost is much lower than Pt. The main drawback of CaCu_3_Ru_4_O_12_ is its resistance to sintering, which was recently overcome by compositing with CuO [14]. Figure 1 shows the temperature dependence of the resistivity of bulk and thick film 20 vol % CuO-mixed CaCu_3_Ru_4_O_12_, which shows good conductivity and metallic temperature dependence.

The heater often causes problems in gas sensors. The temperature dependence of resistivity must be metallic for the heater. For a semiconductive heater, Joule heating increases the heater temperature because of the negative TCR. The decrease in resistance would further increased the Joule heating against a constant voltage. This positive feedback loop eventually causes thermal runaway of the devices [17]. In addition, local defects, compositional deviation, and oxygen deficiency create hotspots [18], which deteriorate and break the heater. Solving these problems is a significant challenge.

In this study, we have constructed a semiconductor-based resistance gas sensor using CuO-mixed CaCu_3_Ru_4_O_12_ as both electrodes and a heater to examine the potential of CaCu_3_Ru_4_O_12_ as a substitute for Pt. We report on the sensor fabrication process, the heater performance, and the sensing performance from the perspectives of chemical and thermal stability of the device. We find the device comprising only ceramics works well and propose that the all-ceramic gas sensor can be widely used.

## 2. Experimental 

### 2.1. Preparation of CaCu_3_Ru_4_O_12_-Based Conducting Paste

CaCu_3_Ru_4_O_12_ was prepared by a solid-state reaction. Stoichiometric mixtures of CaCO_3_, CuO, and RuO_2_ were pressed into pellets and calcined in air at 1000 °C for 48 h. The pellets were covered by a mixture of excess CaCO_3_, CuO, and RuO_2_ powders to prevent Ru sublimation and consequent compositional deviation. The CaCu_3_Ru_4_O_12_ powder was obtained via mechanical grinding and ball milling of the calcined pellets. 

The CaCu_3_Ru_4_O_12_-based conducting paste was comprised of a mixture of pre-synthesized CaCu_3_Ru_4_O_12_ and CuO powders, a vehicle of butyl di glycol acetate (BDGAC) and a commercially available dispersant (DISPERBYK-111, BYK, Wesel, Germany). The volume fraction of CuO in the mixture was 30 vol %, corresponding to 29.5 wt %.

### 2.2. Fabrication of All-Ceramic Gas Sensor

The CaCu_3_Ru_4_O_12_-based conducting paste was printed on an Al_2_O_3_ substrate (3.0 × 25 × 0.3 mm) in a conventional meandering heater shape with 120-μm gaps and 300-μm line width using a screen printing process. Screen printing was performed at the print speed of 50 mm/s using a 500-mesh screen. After drying the vehicle at 120 °C for 1 h, sintering was performed at 1000 °C for 48 h in air. Subsequently the paste was printed on the reverse side of the substrate in a comb-type electrode shape with 120-μm gaps and 100-μm line widths using the same printing process. Heat treatment identical to that used on the heater was performed.

**Figure 2:**
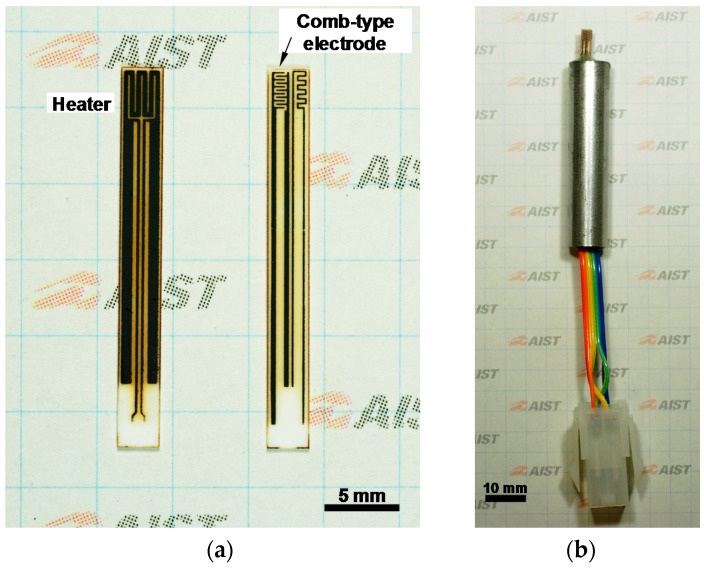
Photographs of (**a**) the heater and electrode sides of the all-ceramic gas sensor and (**b**) the completed sensor package.

We used SnO_2_ particles carrying 1 wt % Pt, 1 wt % Pd, and 1 wt % Au nanoparticles as the sensing material [19]. The SnO_2_ particles were mixed with terpineol and an appropriate dispersant to form a paste, which was hand-painted using a needle onto the comb-type electrode. The all-ceramic gas sensor was obtained after drying the organic components and sintering at 500 °C for 12 h in air. Figure 2a shows photographs of both sides of the obtained sensors. The two thin lines in the center of the heater side and the thin meandering line on the right side of the electrode are the lines for a four-terminal resistance-temperature control and a thermometer, respectively. The SnO_2_ film is invisible in the photograph because of its low thickness.

The substrate was fixed in a stainless-steel tube using epoxy resin after connecting to leads using solder. The completed prototype sensor package is shown in Figure 2b. 

### 2.3. Evaluation of Materials and Sensor

X-ray diffraction (XRD) of the all-ceramic gas sensor was performed using a standard diffractometer with Cu Kα radiation in the 2*θ*-*θ* scan mode (Rigaku SmartLab, Tokyo, Japan). The morphology and element mapping of the sensor were observed using a scanning electron microscope (SEM; JEOL JSM-5600, Tokyo, Japan) and energy-dispersive X-ray spectrometry (EDX; JEOL EX-54145JMU, Tokyo, Japan). The heating characteristics of the heater and temperature distribution of the sensor were measured with a thermal imaging camera (CHINO CPA-T420A, Tokyo, Japan). The coefficient of thermal expansion (CTE) was measured by a thermomechanical analyzer (TMA; Rigaku Thermo Plus EVO2, Tokyo, Japan).

The sensor module was placed in a gas-flow stainless-steel chamber of 530 mL in volume and sealed using ferrules. The sensor module was heated to 400 °C using the CaCu_3_Ru_4_O_12_-based heater under an applied constant voltage. 

Gases for analysis were prepared by mixing N_2_, O_2_, and 1 vol % H_2_ in N_2_ by controlling the flow rates of each gas using a mass-flow controller system. The total gas flow rate was maintained at 1000 mL/min, and the O_2_ concentration was fixed at 20 vol %, or 200 mL/min. We changed the H_2_ concentration in the mixed gas from 200 to 1000 ppm by controlling the flow rate of N_2_ and 1 vol % H_2_ in N_2_.

## 3. Results and Discussion

### 3.1. Chemical Stability of CaCu_3_Ru_4_O_12_

**Figure 3:**
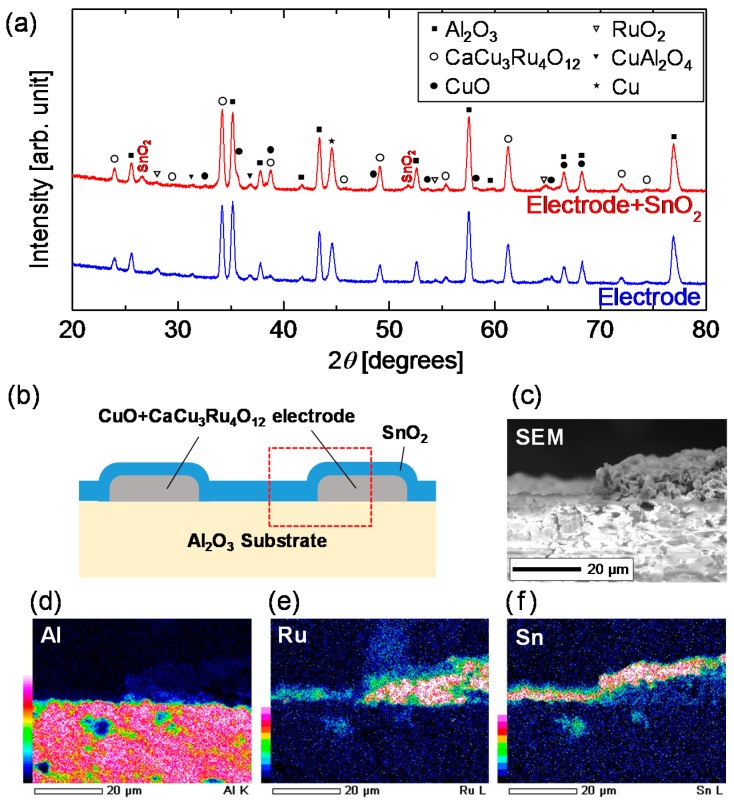
(**a**) XRD (Cu K*α*) patterns of the calcined electrode before and after SnO_2_ coating; (**b**) Schematic of the cross-section of the electrode side; (**c**) SEM image of the electrode side; (**d**–**f**) Elemental mapping for Al, Ru, and Sn, respectively.

Figure 3a shows the XRD patterns of the electrode side before and after SnO_2_ coating. Both measurements were performed after the calcination of each component. In both patterns, the peaks are assigned to Al_2_O_3_, Cu, CuO, CaCu_3_Ru_4_O_12_, and SnO_2_ with tiny traces of RuO_2_ and CuAl_2_O_4_. A substantial amount of Cu is observed, possibly generated from CaCu_3_Ru_4_O_12_ and/or CuO through reduction and decomposition during high-temperature sintering at 1000 °C. CaAl_2_O_4_ is a chemical product of the reaction between the conducting oxide and substrate; this reaction may strengthen adhesion between these components. The only difference between the two patterns is the presence of peaks corresponding to SnO_2_.

Figure 3b shows a schematic of the device, and Figure 3c–f show the SEM-EDX observation images of the electrode side. The observation images correspond to the region enclosed in the red dashed square in Figure 3b. In Figure 3f, Sn is continuously coated on the electrode and the substrate, even at the edge of the electrode. The thickness of SnO_2_ cannot be measured exactly by the Sn mapping because the mapping includes signals from the depth direction. As estimated from Figure 3c, the SnO_2_ thickness seems to be far less than 1 μm. 

### 3.2. Thermal Stability of CaCu_3_Ru_4_O_12_

**Figure 4:**
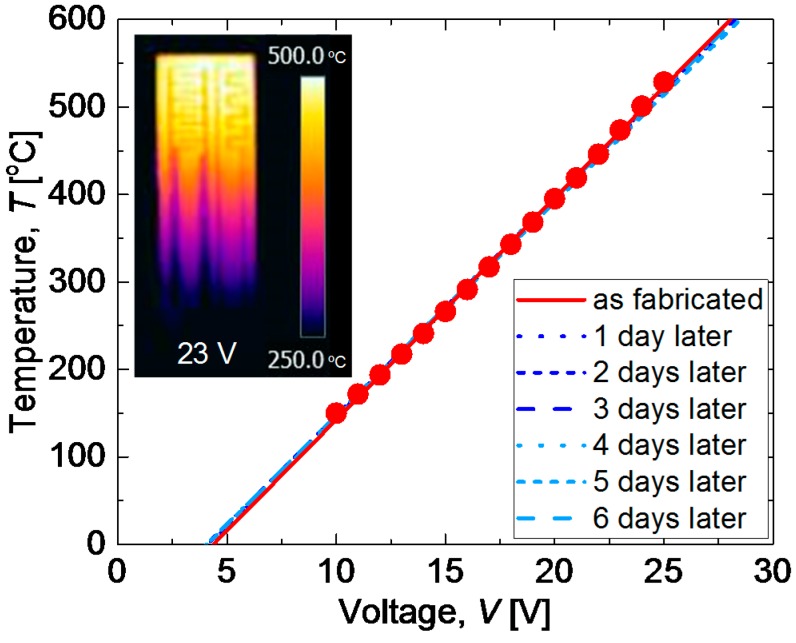
Sensor temperature (*T*), defined as the average temperature of the comb-type electrode, in air as a function of the voltage (*V*) applied to the heater. The closed circles represent the measured data just after fabrication. Solid line is linear fitting result for the closed circles. Dashed lines are fitting lines to the measurement results of each day after maintaining the voltage application of 23 V or more for 24 h.

Figure 4 shows the sensor temperature (*T*), defined as the average temperature of the comb-type electrode, in air as a function of the voltage (*V*) applied to the heater. The closed circles represent the measured data just after fabrication. The temperature increases linearly with the applied voltage, which is expressed as *T* = 25.35*V* − 110.9 in units of degrees Celsius. The inset in Figure 4 is a thermal camera image of the electrode side of the sensor under 23 V applied to the heater, confirming that the comb-type electrode is uniformly heated. To confirm the heater stability for long-term operation, the same measurements were repeated after maintaining the voltage application of 23 V or more for 24 h. This verification was performed for six consecutive days. The dashed lines are fitting lines to the measurement results of each day. Obviously, the responses are highly reproducible, indicating that the heater remains intact for long-term operation at high temperatures. 

**Figure 5:**
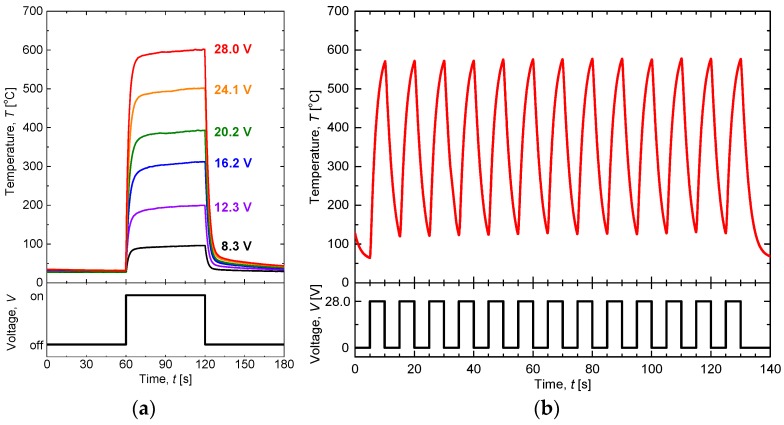
(**a**) Temperatures of the sensor under single-pulse voltage of 60 s in width and various voltage amplitudes applied. (**b**) Temperature of the sensor under cyclic 28.0 V pulse with width of 5 s and cycle time of 10 s. Both measurements are performed in air.

To examine the quick response and durability in air, we investigated the heater response against pulsed voltage of 60 s. Figure 5a shows the sensor temperature with single pulses of 60 s, in which the pulse voltages varied from 8.3 to 28.0 V. At all applied voltages, the temperatures change rapidly with the pulse, reaching 90% or more of the final temperature within 15 s of the start of the pulse. The temperatures continue to rise gently at the end of the pulse. This is simply because the temperatures have not yet reached the steady state because of structural factors. Defining the relaxation time as the time to reach 90% of the temperature at the end of the pulse, the longest is 8.5 s at 12.3 V. The temperature of the sensor under a 28.0 V pulse with a width of 5 s and a cycle length of 10 s is shown in Figure 5b. The heater is robust against a large temperature change of 500 °C within 10 s. 

The heater performance is most important in the operation of high-temperature gas sensors. In particular, the heater controllability and stability are related to the convenience and reliability of the sensor. The results shown in Figure 4 and Figure 5 demonstrate the quality of the heater. The CTE of 30 vol % CuO-mixed CaCu_3_Ru_4_O_12_ bulk was measured to be ~8.2–8.7 × 10^−6^/K, which is close to that of commercial Al_2_O_3_ at ~7–8 × 10^−6^/K. This CTE matching is one reason underlying the stable high-temperature operation of the heater. A thermal cycle test is usually performed such that the devices are heated and cooled over a relatively long time span of 100–200 s [20], which is much gentler than that shown in Figure 5b. We therefore conclude that the 30 vol % CuO-mixed CaCu_3_Ru_4_O_12_ thick-film heater is surprisingly robust and reliable and can replace Pt heaters in gas sensors. 

### 3.3. Demonstration of All-Ceramics Gas Sensor

**Figure 6:**
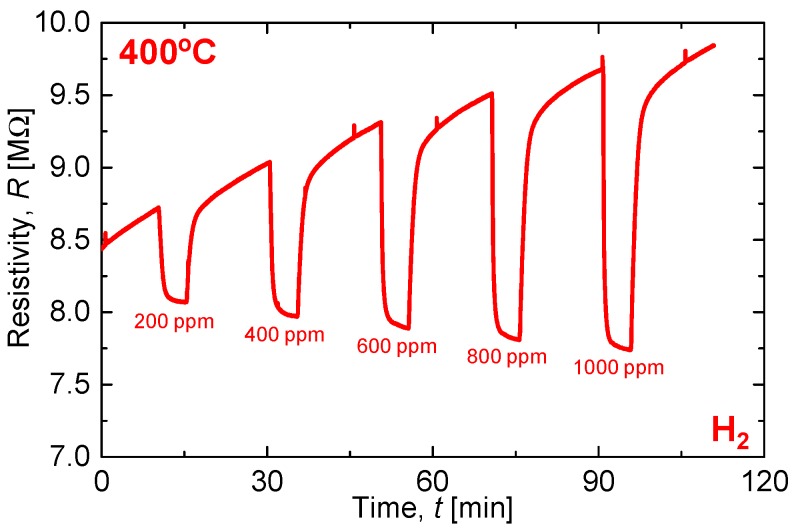
Sensor resistance change under various H_2_ concentrations at 400 °C.

Figure 6 shows the sensing performance of the all-ceramic gas sensor under various H_2_ concentrations. The measurements are performed at the sensor surface temperature of 400 °C, heated using the 30 vol % CuO-mixed CaCu_3_Ru_4_O_12_ heater on the back side of the sensor substrate. The resistance is in the order of several mega-ohms, four orders of magnitude higher than the electrode resistance of hundreds of ohms. Therefore, the measured resistance is mostly that of the SnO_2_ sensing material. The sensor resistance decreases during H_2_ flow and the response increases with increasing H_2_ concentration. Gas-sensitive metal oxide of SnO_2_, are porous thick films of particles. In air, pre-adsorbed chemical species and O_2_ molecule adsorption makes the surface of particle oxidized with the charge carrier of an electron, so that the electron on the surface of particles is depleted and of high resistance. When H_2_, the surface is reduced, or H_2_ is oxidized, so that the electron is released into the particles and of low resistance. As the H_2_ concentration increases on the electron, it seems that gas molecules play roles of electron donors and SnO_2_ sensing material accepts additional electrons. Because of the drift in the base resistance, which is the resistance in air (*R*_a_), the exact response value cannot be calculated. However, the response is roughly proportional to the H_2_ concentration. The sensor response *S* can be expressed as *S* = *R*_a_/*R*_g_ using the sensor resistance under the target gas flow *R*_g_. From Figure 6, *S* for 1000 ppm H_2_ is calculated as 1.25. For the same material under less reducing gas, *S* can exceed 30 under optimal conditions, in which the thickness of the sensing material is 3 μm and the sensor temperature is 250 °C [19]. The very small *S* value in this study is because the device structure is not optimized. The response time of 90% of saturated level, *t*_90_, of the sensor was slightly dependent on H_2_ concentration, 1.3 min for 200 ppm and reduced to 0.81 min for 1000 ppm, which are common behaviors of a SnO_2_ gas sensor. The recovery time is about 2–3 min, which is fairly fast; however, it is not fast in detail because of the drift of the resistance in air. This *t*_90_ is originated fundamentally from the kinetics of gas adsorption on the SnO_2_ surface so that there was no difference between CuO-mixed CaCu_3_Ru_4_O_12_ and Pt lines. We also notice that the base resistance increases over time because of the short wait time; a long warm-up time is necessary to remove atmospheric volatile organic compounds adsorbed on the sensing material surface. To achieve the best performance, the device structure should be optimized. Nevertheless, we conclude that CuO-mixed CaCu_3_Ru_4_O_12_ works well as both a heater and electrode and that the trial of all-ceramic gas sensor detects H_2_ reasonably well. 

## 4. Conclusions

We have fabricated a semiconductor-based resistance gas sensor using a heater and electrodes comprising of 30 vol % CuO-mixed CaCu_3_Ru_4_O_12_ to demonstrate the potential of CaCu_3_Ru_4_O_12_ as a Pt substitute. The sensor was completed as designed using a screen-printing process, and the results of XRD and microstructure observations supported the fabrication and design from perspectives of material and structure. The 30 vol % CuO-mixed CaCu_3_Ru_4_O_12_ heater realized a working temperature exceeding 600 °C by Joule heating, with linear increases in temperature with respect to the applied voltage. The heater remained intact after long-term operation at high-temperature and a large temperature change of 500 °C within 10 s. A possible reason for this robustness is the good matching of the CTE between the 30 vol % CuO-mixed CaCu_3_Ru_4_O_12_ thick film and the substrate. The sensing performance of the sensor to H_2_ was similar to that of a conventional semiconductor-based resistance gas sensor. The performances of the heater and sensor indicate that CaCu_3_Ru_4_O_12_ is a reliable conducting material that can replace Pt in various devices.

## Figures and Tables

**Table 1:** Electrical resistivity at 500 °C (*ρ*_500_), TCR (*α*_30–500_), and material cost of Pt and CaCu_3_Ru_4_O_12_ bulk [10].

Material	*ρ*_500_ (μΩ∙cm)	*α*_30–500_ (%/°C)	Cost ($/kg)
Pt	27.5	0.324	50,000
CaCu_3_Ru_4_O_12_	937.4	0.135	950
